# Supplementary material for: Local field potentials primarily reflect inhibitory neuron activity in human and monkey cortex
Source: Sci Rep. 2017 Jan 11;7:40211. doi: 10.1038/srep40211 (PMC5225490; doi:10.1038/srep40211)
Supplement: Supplementary Information [file srep40211-s1.pdf]

Supplementary Information to "Local field potentials primarily reflect inhibitory neuron activity in human and monkey cortex"

Teleńczuk B, Dehghani N, Le Van Quyen M, Cash SS, Halgren E, Hatsopoulos NG, Destexhe A

1 Supplementary Methods

1.1 Validation of the whitening technique

To validate the whitening method and, specifically, to show that it recovers the post-synaptic contribution to the LFP from a spike of a single neuron (the unitary LFP), we tested it on a model LFP signal. The LFP is modelled as a linear superposition of the trans-membrane currents generating the LFP (LFP sources).

For simplicity, we test the method on LFP signals recorded using only two electrodes, but this approach can be extended to multi-electrode arrays (Utah arrays). The LFP sources are modelled as post-synaptic currents triggered by a population of neurons. The contribution of these current to the LFP signal is quantified as the spatio-temporal LFP kernel, $k(t-t', x-x')$, where $x-x'$ is the distance of the electrode from the spiking neuron and $t-t'$ is the time from a spike. LFP source at given position x is then calculated as a sum of such kernels centered on all neurons and all spikes:

$$s_x(t) = \sum_i \sum_t k(t - t'_{ik}, x - x_i)$$

where t'_{ik} $(k = 1 \dots K)$ are K spikes of neuron i, x_i is the position of neuron i.

The contribution from the local population decays exponentially in time and space, $k_l(t-t', x-x') = \exp((t-t')/\tau)\exp((x-x')/\lambda)$ for $t > 0$, $k_l(t-t', x-x') = 0$ otherwise; the contribution of remote population is constant in space, $k_r(t-t', x-x') = \exp((t-t')/\tau)$. The rationale for the assumption is that the probability of connections between neurons decays very fast in the close neighbourhood of the neuron, but for distances above 1 mm it stays at small and constant level (Peyrache et al. 2012). The size of the remote population is large (it covers much larger area that the local population within the radius of < 1 mm), so that it's contribution can be significant, even if single neurons contribute little. Alternatively, the contribution from far population can be indirect, i.e., mediated by common inputs or modulations of excitability rather than direct connections.

We generate spikes of local population with constant rate using Bernoulli process. The spikes of remote population are modelled using Poisson process. The contributions to the LFP sources of

both populations are summed together. Finally, we obtain the LFP by a linear superposition of the sources using a mixture matrix, L, with elements $l_{xx'}$ (also called a lead matrix in the EEG literature). This mixing operation is often referred to as the volume conduction. Using Einstein summation notation it can be represented as following:

$$\mathrm{LFP}_x(t) = l_{xx'} s_{x'}(t)$$

Note that for simplicity we assumed linear, homogeneous and ohmic medium, but it's possible to generalise to non-ohmic media.

We estimate the LFP kernel using only the simulated LFP signal and spikes of local population. We calculated the spike-triggered LFP (st-LFP) the standard way (see Methods) and compare it with the original LFP kernel (which normally would not be available to the experimentalist). We found that the st-LFP is broader spatially than the kernel, i.e. its amplitude decays slower with distance than the model LFP kernel (Supplementary Figure 1, left). This broadening is due to passive spread of electric field (the volume conduction), which is parametrised by the mixing (lead) matrix.

To recover the LFP kernel we might apply a spatial filtering. The optimal spatial filter is given by the inverse of the mixing matrix, $U = L^{-1}$, with elements $u_{xx'}$.

$$\hat{k}(x,t) = u_{xx'} \text{st-LFP}_{x'}(t)$$

Application of this filter to the st-LFP allows to recover the LFP kernel with the same spatial and temporal dependence (Supplementary Figure 1B):

In practice, we do not know the mixing matrix, so we can not estimate the optimal unmixing filter. Therefore, we resort to the whitening technique, which allows to estimate the best filter directly from the data. To this end, we use a regularised pseudo-inverse of the ongoing LFP covariance matrix, W (see Methods). We find that this technique allows to recover the LFP kernels, which are very close to the ones obtained using the optimal unmixing matrix, but also the original LFP kernel (Supplementary Figure 1C).

2 Supplementary Figures

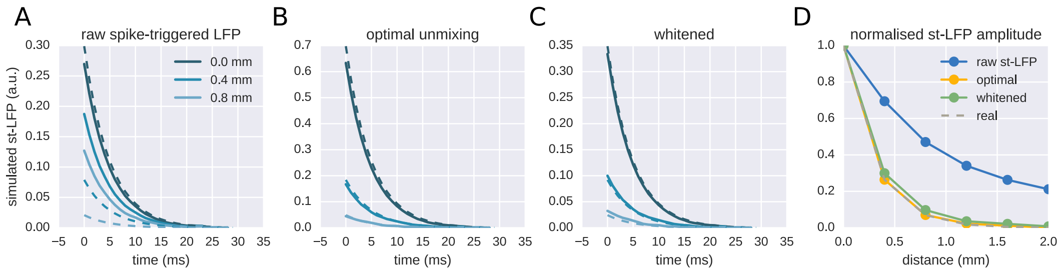

Supplementary Figure 1: Comparsion between the modelled LFP kernel (dashed line in all panels) and kernel estimated using three different methods (solid lines): standard st-LFP (A), optimal un-mixing using known mixing matrix (B), whitening (C). Color variations represent distance from the trigger neuron. The whitened st-LFP optimally recovers the amplitude decay of the kernel (D)

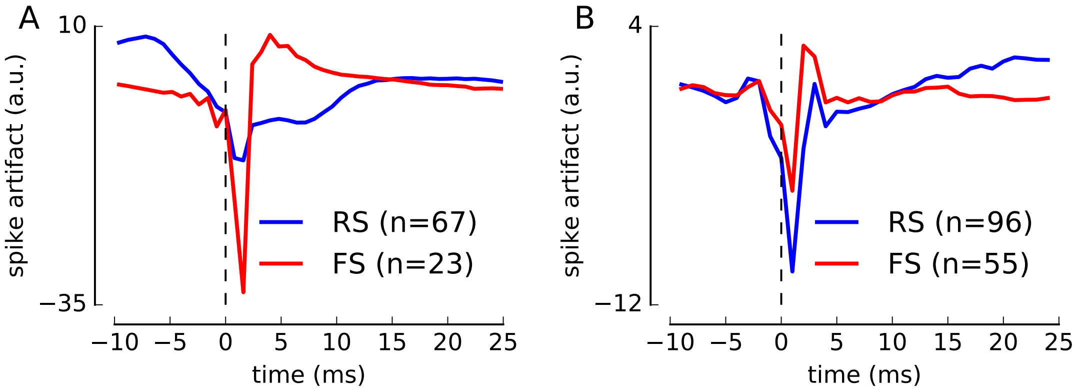

Supplementary Figure 2: The st-LFP for the electrodes where the spikes of each neuron were identified, averaged across all neurons of specific type (regular spiking, RS, or fast spiking, FS). (**A**) Human temporal lobe. (**B**) Monkey pre-motor cortex. The sharp peaks represent spike artifacts. To avoid the contamination of the whitened st-LFP these electrodes were removed prior to the whitening.

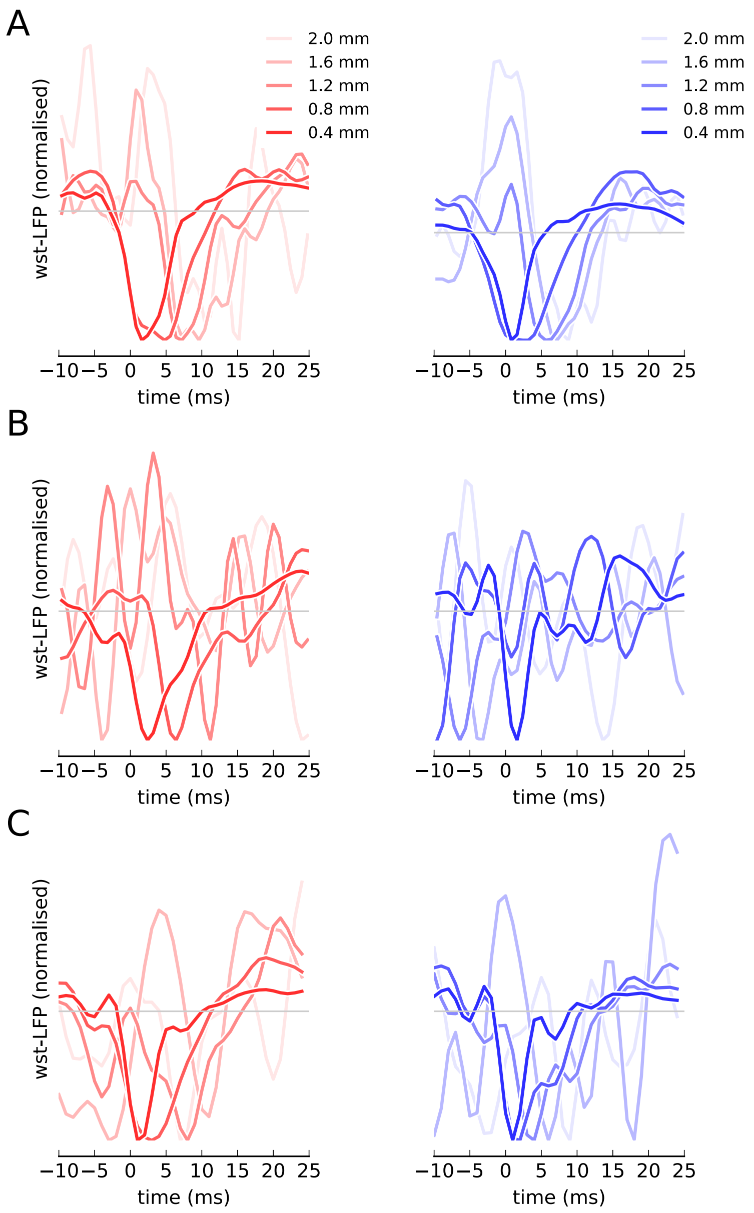

Supplementary Figure 3: The whitened st-LFP traces at constant distances from the trigger neuron for FS (left, red) and RS (right, blue) neurons. The shade of the curve represents distance (see legend). (**A**) Human subject 1. (**B**) Human subject 2. (**C**) Macaque monkey.

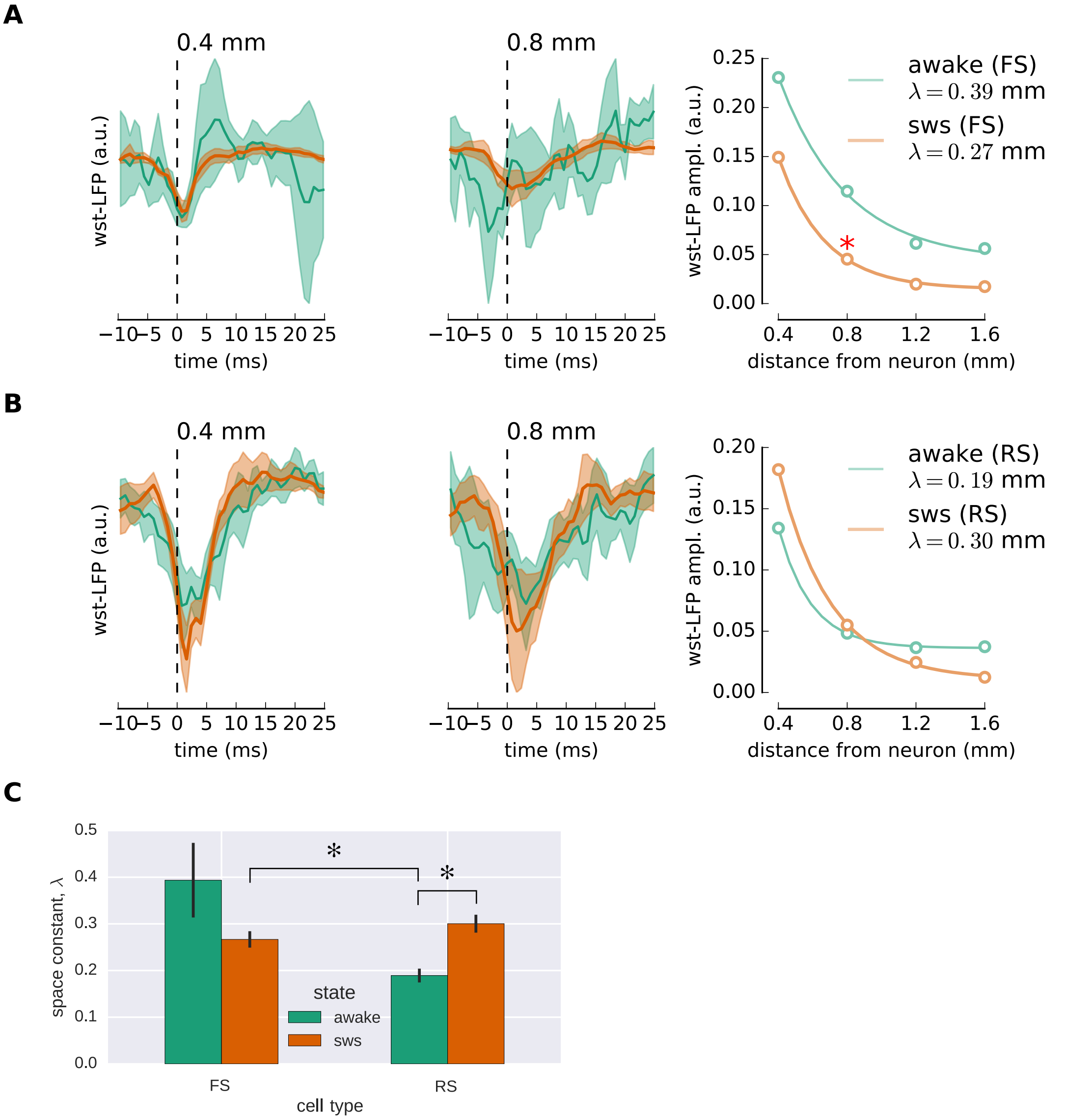

Supplementary Figure 4: Comparison of wst-LFP for two states (awake, green, and slow-wave sleep, orange) in human subject 1. (**A**, **B**) wst-LFP (solid line) was averaged across of FS (A) and RS (B) neurons at 0.4 mm (left) and 0.8 mm (right) from the neuron. The shaded area represent 95% confidence intervals (calculated as mean ± 1.96 s.e.m.). The decay of wst-LFP amplitude with distance is shown in right-most panels. The red star denotes significant difference in amplitudes for the particular distance (bootstrap test, $p<0.05$). (**C**) Comparison of the space constants across states and neuron types. The error-bars represent the standard deviation of fitted coefficient. The bracket point to significant differences betweent the space constants (t test, $p<0.05$).

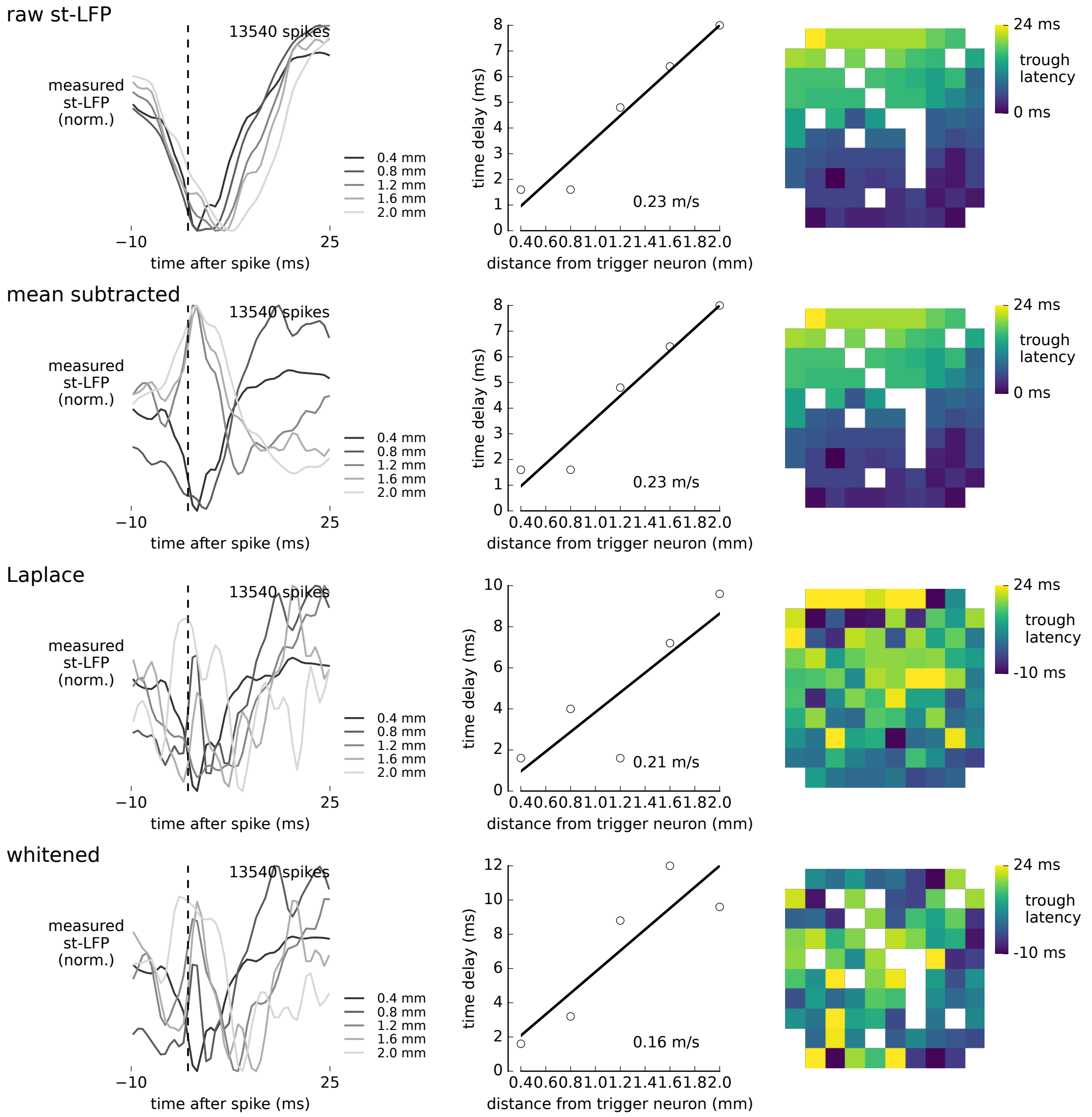

Supplementary Figure 5: Comparing of the st-LFP calculated from a single cell of the human subject 1 with the st-LFP filtered spatially using four different methods: subtraction of mean of all channels, applications of discrete Laplace operator and spatial whitening. Note that subtracting mean is equivalent to using averaged reference.

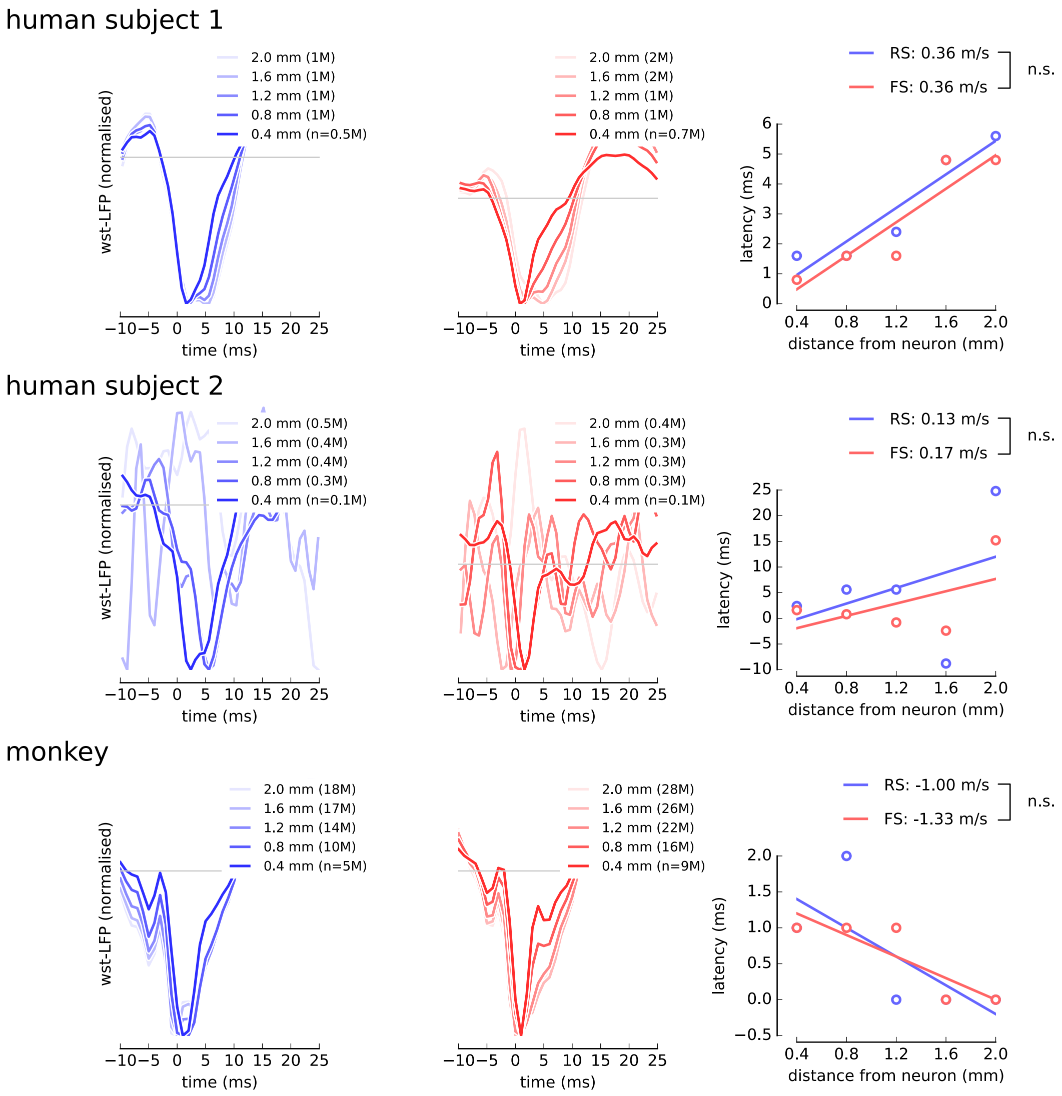

Supplementary Figure 6: Propagation of non-whitened st-LFP. *Left/Middle*: Averaged st-LFP traces at constant distance from the trigger neuro that were used to calculate the latencies shown in the right panel. *Right*: Latency vs distance (circle) fitted by a linear function (solid) line for each neuron type. The propagation speeds (at the top) were calculated as the inverse of the linear slope. Differences between the speeds for RS (blue) and FS (red) neurons are tested using bootstrap method.

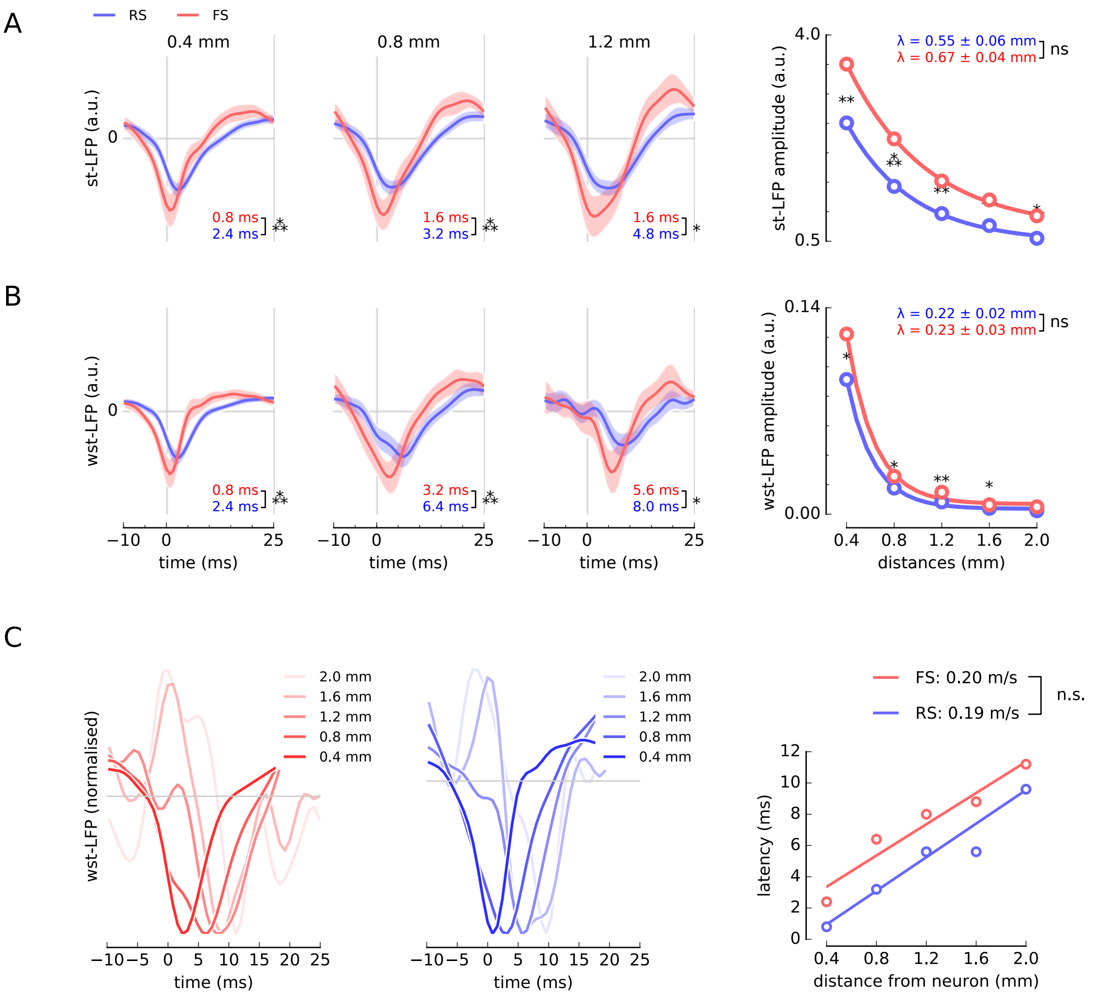

Supplementary Figure 7: Spike-triggered LFP for the second night of human subject 1. (**A**) non-whitened st-LFP amplitude vs. distance. For detailed legend see caption of Figure 2. The stars above the amplitude values in the rightmost panel represent the p-value of differences in st-LFP amplitudes between FS (red) and RS (blue) neurons. (**B**) Same as (A) but for whitened st-LFP (wst-LFP). (**C**) Comparison of wst-LFP at increasing distaces from trigger neuron for FS neurons (left) and RS neurons (right). The rightmost panel shows the increase of through latency with distance (dots) and estimation of propagation speed (lines, for values of estimated speed see legend above). The results are consistent with the results obtained in the first night of recording in the same patient (compare with Figures 2A, 3D and 4B). n.s: not significant, $*$: $p < 0.05$, $**$: $p < 0.01$, $***$: $p < 0.001$

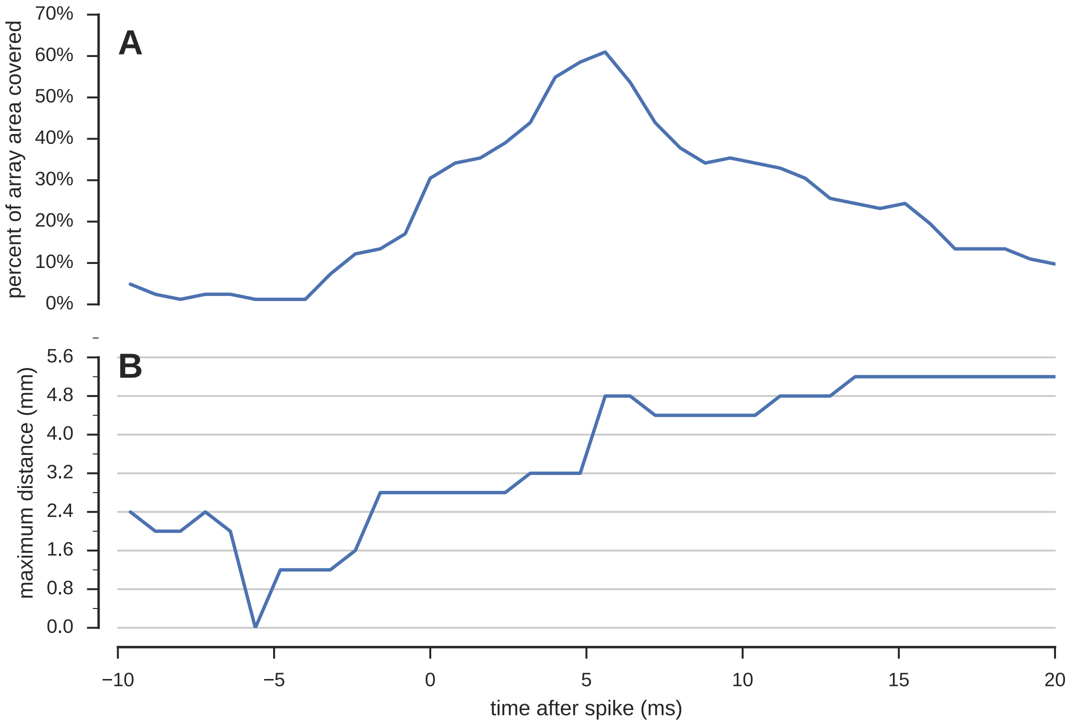

Supplementary Figure 8: Spatial range of the single-neuron st-LFP deflections that are significantly more negative than the noise level (gray area in Figure 1C, bottom panel) shown across time. (**A**) Fraction of electrodes with such significant st-LFPs across different time points. (**B**) The distance between the neuron and the furthest electrode with significant st-LFP. The electrode furthest from the trigger neuron is at 5.2 mm; the maximum distance between any pair of electrodes is 6.4 mm.

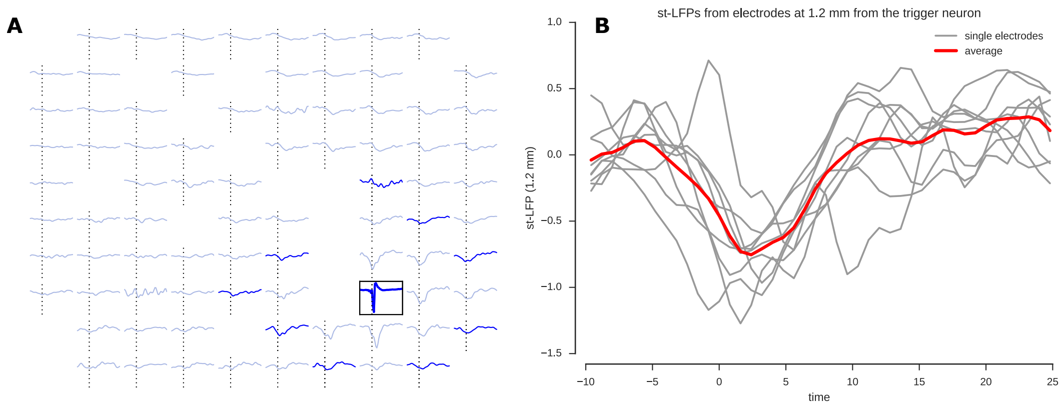

Supplementary Figure 9: Averaging of st-LFPs across electrodes with the distance of 1.2 mm from the trigger neuron. (**A**) st-LFPs of all electrodes triggered on spikes of a single neuron (black square). The st-LFPs 1.2 mm away from the trigger neuron (Manhattan distance) are shown in dark blue. Note that the Manhattan distance implies that the constant-distance contours form a square rotated by 45°. (**B**) The selected st-LFPs shown on the same axis (gray lines) together with their average (red, same as the right panel of Figure 1D in main text). Small negative deflections survive the averaging whereas other fluctuations only present in single electrodes are averaged out.

3 Supplementary Tables

Supplementary Table 1: Summary of number of neurons and spikes used in the calculation of spike-triggered LFP for each subject. Neurons with less than 1000 spikes were removed from the analysis.

	**number of neurons**	**number of spikes**			
		total	median	min	max
Human subject 1 (first night)					
FS neurons	20	269 680	7 517	1 432	66 314
RS neurons	49	163 758	2 376	1 015	13 540
all neurons	69	433 438	2 753	1 015	66 314
Human subject 1 (second night)					
FS neurons	17	194 071	12 310	1 292	32 667
RS neurons	37	143 300	2 924	1 098	19 549
all neurons	54	337 371	3 174	1 098	32 667
Human subject 2					
FS neurons	4	40 725	8 265	1 075	23 120
RS neurons	13	51 210	3 311	1 243	16 735
all neurons	17	91 935	3 311	1 075	23 120
Monkey					
FS neurons	55	2 089 180	20 711	2 225	191 230
RS neurons	95	1 466 565	11 998	1 089	65 335
all neurons	150	3 555 745	13 715	1 089	191 230

Supplementary Table 2: Firing properties of neurons used in the analysis averaged across neuron of RS/FS types. To avoid contribution from prelonged silences we calculated the statistics in 5-minute-long intervals, which contained at least 2 spikes. SD = standard deviation, SEM = standard error of mean, min/max = minimum and maximum over neurons.

	**firing rate, Hz**					**coefficient of variation**				
	mean	SEM	SD	min	max	mean	SEM	SD	min	max
Human subject 1 (first night)										
FS neurons	2,63	0,56	2,55	0,43	8,20	2,44	0,21	0,95	1,26	4,81
RS neurons	0,66	0,07	0,51	0,14	2,61	1,88	0,06	0,42	1,33	3,47
Human subject 1 (second night)										
FS neurons	2,16	0,38	1,63	0,29	4,82	1,94	0,06	0,27	1,36	2,33
RS neurons	0,66	0,08	0,51	0,13	3,00	1,68	0,05	0,28	1,30	2,46
Human subject 2										
FS neurons	0,77	0,30	0,68	0,16	1,58	2,02	0,36	0,84	1,42	3,26
RS neurons	0,32	0,10	0,38	0,08	1,55	1,72	0,08	0,31	1,46	2,62
Monkey										
FS neurons	3,63	0,55	4,15	0,18	21,06	1,78	0,06	0,44	1,09	2,75
RS neurons	1,31	0,11	1,11	0,09	5,96	1,43	0,03	0,26	1,03	2,46

References

Peyrache, Adrien, Nima Dehghani, Emad N Eskandar, Joseph R Madsen, William S Anderson, Jacob A Donoghue, Leigh R Hochberg, Eric Halgren, Sydney S Cash, and Alain Destexhe. 2012. "Spatiotemporal Dynamics of Neocortical Excitation and Inhibition During Human Sleep." *Proc. Natl. Acad. Sci. U.S.A.* 109 (5): 1731–6. doi:10.1073/pnas.1109895109.
